# Welfare paternalism and the paradox of protection: towards a rights-based framework to mediate the deprivation of liberty of children

**DOI:** 10.1093/medlaw/fwag029

**Published:** 2026-08-14

**Authors:** Clayton Ó Néill

**Affiliations:** School of Law, Queen’s University Belfast, Belfast BT7 1NN, United Kingdom

## Abstract

This article examines the acute ambiguity in the law governing the deprivation of liberty of children in England and Wales, where protective interventions often rely on judicial direction and on unclear statutory safeguards. It is argued that the current framework is reactive, welfare-driven, and lacking in procedural coherence. Drawing upon Fineman’s theory of vulnerability, Fraser’s parity of participation, and Lundy’s analysis of children’s agency in relation to the participatory guarantees of Article 12 of the United Nations Convention on the Rights of the Child, the article reconceptualizes vulnerability and rejects the paternalistic protection ethos of the status quo. The article exposes structural gaps in the light of a shifting legal landscape. This backdrop includes the Supreme Court’s recent 2026 move away from the rigid, single-determinant tests for confinement towards contextual, multifactorial assessments. It proposes an original statutory architecture: the Children’s Liberty Protection Framework (CLPF) that establishes statutory mechanisms of advocacy, review, and oversight at Tribunal level. The CLPF transforms vulnerability from being a control-based condition into a procedural right to participate. As such, it offers a coherent legal pathway for reconciling the challenges posed by the right to liberty and the need to protect children within a supportive and responsive state.

## Introduction

In England and Wales, children’s freedom is constrained every day by systems meant to protect them. Consider a scenario in which a child in care is constantly monitored and forbidden to leave the premises without permission. He is told that these limitations are for his safety. In this circumstance, the agency and autonomy of the child are diminished, often without appropriate or coherent safeguards. A paradox exists: how can we ensure the child’s safety while respecting his right to participate in decisions that have a profound impact on his life? This paradox of protection relates to the tension that exists where measures are introduced to protect the best interests of a child while, at the same time, these measures restrict his or her liberty and emerging developing autonomy. This raises questions about when care that is designed to be protective in nature enters into the realm of rights-infringing confinement. In situations where there is a risk to the child or evidence of vulnerability or exploitation, it will be seen that protective interventions can promote and weaken the rights that are afforded to the child. The current legal framework lacks conceptual clarity and, at times, the law can struggle to recognize whether restrictions placed on a child result in a deprivation of liberty (DoL) and on what basis there can be justification for such restrictions. The structural ambiguity in relation to children differs from the regime for adults. Under the Mental Capacity Act 2005 (MCA), the Deprivation of Liberty Safeguards (DoLS) were introduced. The aim of the DoLS was to provide procedural safeguards for adults who lacked capacity and were deprived of liberty in care environments or hospitals. Under the DoLS, there is a need for assessment, authorization, review, and right of challenge. It must be noted that, even though they do not apply to children and will be replaced by the Liberty Protection Safeguards (LPS), they are still important because they show the type of statutory framework that does not exist for children. As a consequence, children can be subjected to a DoL through different avenues, without an equivalent dedicated statutory scheme. The LPS, introduced under the Mental Capacity (Amendment) Act 2019 in England and Wales, were introduced to replace the DoLS and to provide a more coherent framework governing DoL for people who lack capacity. The safeguards extend to 16- to 17-year-olds. The LPS have, however, been criticized and their implementation has been delayed.^1^ Critique of the scheme has been based on the complexity of the framework, the potential reduction of independent examination and the degree to which aims that are focused on efficiency may override robust rights protections.^2^ Questions arise, also, as to whether the safeguards will actually support meaningful participation or risk being merely procedural or formalistic. There has, therefore, been an effort to construct procedural pathways to regulate the confinement of adults; the DoL of children is governed by a fragmented framework that lacks statutory independent oversight. This is partly because children are, by their nature, supervised and controlled by parents or guardians.

It must be noted, however, that the environments in which children navigate their world, including school and educational settings, are already structured and have supervisory elements. There is an associated difficulty in recognizing when restrictions go too far and when restrictions cross the threshold into something more significant. In particular, it is not always clear when restrictions exceed what would normally be regarded as appropriate for another child of the same age and similar development. Individuals in restrictive settings experience a significant loss of ‘decision-space’.^3^ In such contexts, micro-level decisions about routines and personal activities are often under the control of carers and staff. The logistics of institutional routines primarily govern everyday life.^4^ There is, thus, a related risk that formal rights have little practical effect if a mechanism is not in place to address the hidden dynamics of control.

It is argued here that the current regime for children ought to place less emphasis on pure welfare paternalism and should instead give greater attention to supported decision-making and proportionality. It is also contended that the approach to children should be more closely aligned with that of adults, while still maintaining a child-oriented focus. The article questions whether a rights-oriented framework can reconcile children’s liberty with the protective interventions necessary under Article 5 of the European Convention on Human Rights (ECHR). It also proposes a new statutory framework to replace existing mechanisms: the Children’s Liberty Protection Framework (CLPF). A model of statute is presented here that is autonomy-maximizing, while also protecting children and promoting procedural justice.^5^ The CLPF is aligned with vulnerability theory. This theory is an appropriate starting point for understanding the structural conditions that influence the lives of children. It is connected to a relational and developmental version of autonomy (in order to prevent placing too much emphasis on paternalistic approaches). Thus, the CLPF is presented as a model where children are regarded as rights-holders, which include both rights to participation and protection. Paternalistic interventions may be defensible within a framework that gives weight to children’s developing capacities.

As it stands, the law lacks an adequate conceptual mechanism for recognizing when what appears to be restrictive care arrangements have sojourned into the realm of detention.^6^ A shift to a rights-based statutory model is proposed, which links protection with autonomy through supported decision-making, structured participation, and proportionality.

There are two original elements to this article. The article’s first original contribution is to reconceptualize vulnerability not as a justification for paternalistic control but as a procedural foundation for participatory safeguards pertaining to the DoL of children. Secondly, it develops the CLPF, which makes a clearer distinction between the identification of DoL and the question of justification, which is a separate issue. By doing this, it is argued here that the current legal framework gives insufficient protection to children’s participatory rights under Article 5 ECHR and Article 12 of the United Nations Convention on the Rights of the Child (CRC) due to the fact that welfare considerations often collapse these stages, which should be analytically distinct.

### A roadmap towards a new approach

The following structure applies: section ‘Setting the scene: children and deprivation of liberty’ begins by setting the scene regarding children and DoL. The section distinguishes between legal authorization and justification. It introduces the tension between the rights of children and welfare paternalism. This sets up the argument later made in the article for a reframed, rights-oriented approach that is procedurally clearer. Section ‘A fractured system: The Children Act 1989, inherent jurisdiction, and Article 5 ECHR’ sets out the legal framework pertaining to DoL. It focuses on three core areas: the Children Act 1989 (CA), the inherent jurisdiction of the High Court, and Article 5 ECHR. Together, these frameworks point to a broken, reactive approach in which courts are often seen to be compensating for systemic failures in the legal system.

Sections ‘Autonomy, capacity, and the limits of welfare paternalism’ and ‘Vulnerability and parity of participation’ develop the article’s conceptual framework, exploring the roles of paternalism, rights, autonomy, and vulnerability, and the tensions among these concepts in this context. Section ‘Vulnerability and parity of participation’ develops a framework embedded in vulnerability theory, parity of participation, child-centred autonomy, procedural fairness, and voice, and reduces the focus on paternalistic protection. In section ‘From theory to procedure: A case for reform and the proposed children’s liberty protection framework’, reforms are proposed, including a new statutory regime for children, the CLPF, modelled on the approach for adults but with a child-oriented dimension. A new system needs to better align with the CRC and the 2019 Council of Europe Recommendations on migration.^7^ In the Conclusion section, it is contended that we should not view autonomy and protection as clashing but, instead, as complementary. The role of the article is to view the paradox of protection not through the lens of welfare, but as a mechanism for adjudicating rights. An attempt is made to establish an approach that is more conceptually clear than the status quo, and a justifiable basis for the imposition of DoL.

The reality of ‘social care detention’ is most visible in the day-to-day running of contemporary placements. Such settings may appear domestic and benignly familial, but usually encompass continuous supervision, significant limits on decision-making, and constricted exit routes.

Following the decision of the Supreme Court in *A Reference by the Attorney General for Northern Ireland*, there has been a departure from the ‘acid test’ in *Cheshire West.* This test understood DoL as a situation in which a person is under continuous supervision and control and is not free to leave. The Supreme Court has now held that there is no single determinative test for DoL. It was also reaffirmed that for the objective aspect of DoL, there is a need for a multifactorial assessment of the ‘type, duration, effects and manner of implementation’ of the restrictions on liberty imposed.^8^ The key features in *Cheshire West* (continuous supervision/control and the person is not free to leave) remain important factors, but they are not definitive markers of DoL. There is an emphasis on the need for the approach to be ‘practical and realistic’^9^ and that ‘[o]rdinary expectations and the ordinary course of life play a significant role’ in deciding if Article 5 ECHR is engaged.^10^ Any discussion of *Cheshire West* in this article ought to be considered in light of the understanding of DoL between 2014 and 2026 and not as the current legal position. This long-standing model was disrupted by the Supreme Court’s decision, which marks a major turning point for contemporary law on restrictions on liberty.^11^

The decision of the Supreme Court relates to people over the age of 16 years, but its reasoning may play an important role for children. Focusing on context, normality and the lived reality of restrictions on liberty echoes issues that have been previously brought to light about transporting adult concepts into childhood. Also, the judgment recognizes the three elements of Article 5 set out in *Storck v Germany*, namely the objective element (‘the person is confined to a particular restricted space for a period of time’), the subjective element (‘where there is no “valid consent” to that confinement’), and the responsible of the state, directly or indirectly, for the confinement.^12^ Therefore, the Supreme Court has continued to recognize the relevance of questioning issues of consent, parental responsibility and the role of public authorities. The specific impact of this case on younger people remains to be seen, but it is very likely that it will involve an analysis that is more child-focused and context-driven than under the more inflexible approach in *Cheshire West*.

It is worth noting that in *Re D (A Child)*, in relation to children, the issue is more challenging due to age and parental control.^13^ This challenge to the concept of welfare paternalism involves a reconceptualization of vulnerability as a vehicle for a potential reform model. The CLPF focuses on proportionality, the recognition of a child’s developmental stages, and participatory safeguards. It translates the theoretical shift through coherent safeguards. It is inspired by the new (not yet enacted) Liberty Protection Safeguards (LPS) for adults. Therefore, the gap between theory and procedure is bridged. A new framework, which is based on an application of vulnerability theory and connected to a relational and developmental concept of autonomy, sets out when a DoL exists, and it also presents a basis for justifying a deprivation in the case of children.

### The conceptual toolkit: Negotiating autonomy and paternalism

The article addresses a number of core concepts. The concepts of autonomy and paternalism are often invoked in ways that make it difficult to determine their relative weight when analysing the law in this area, resulting in a position where their roles can be uncertain, especially in relation to the justifiability of restricting liberty. For the purposes of this article, autonomy is viewed as developmental and relational.^14^ This reflects the ability of the child to express preferences within supportive frameworks. Relational versions of autonomy regard decision-making abilities as forming in a gradual manner.^15^ From this perspective, society and institutional environments influence the expression of autonomy. It is not about an all-or-nothing version of decision-making. Autonomy is not regarded as something that is binary. The decisions made by children reflect the social environments in which they live, their relationships with others, and their maturity, which change over time. Herring explains that ‘[i]t requires that people’s decisions are understood in the context of the relationships they live in and the obligations that flow from those relationships are given due weight.’^16^ Mackenzie and Stoljar argue that relational autonomy involves a ‘shared conviction… that persons are socially embedded and that agents’ identities are formed within the context of social relationships and shaped by a complex of intersecting social determinants, such as race, class, gender, and ethnicity’.^17^

The second concept is paternalism, which involves approaches that restrict a person’s wishes in line with their best interests.^18^ Gerald Dworkin states that paternalism involves ‘interference with a person’s liberty of action justified by reasons referring exclusively to the welfare, good happiness, needs or values of the person being coerced’.^19^ According to Feinberg, the paternalistic perspective means that ‘it is always a good reason in support of a… prohibition that is necessary to prevent harm (physical, psychological, or economic) to the actor himself’.^20^ Paternalistic intervention is also seen as the enemy of autonomy, but, when it comes to children, there is an inevitability to some level of paternalism which, in many cases, is justified, even for those who take a strong perspective on the manifestation of autonomy. What is relevant here is under what conditions paternalistic intervention is defensible. When a child’s capacities are developing, interventions that are in line with their best interests may actually promote their future autonomy. These types of measures might be justified by a need to respect the emerging capacities of the child and not to disproportionately interfere with their freedom.

Rights are not regarded as a single unified category. A distinction is made between rights to self-determination and rights to protection. Tension is created by the need to reconcile these competing dimensions. Regarding children as rights-holders does not mean that paternalism does not play a role. Paternalistic interventions must be justified in relation to best interests and also whether they are compatible with the child’s rights. Defining paternalism as treating children as passive subjects of care depends upon how the rights that are at stake are understood. If children’s rights include rights to protection and welfare, then certain paternalistic interventions may actually be required by those rights. In the same way, when paternalism is attentive to the child’s wishes, feelings and values, then the child is not passive, but is an active part of the decision-making process. The critical issue is, therefore, not the presence of paternalism, but whether it is exercised in a way that is sensitive to both the protective and participatory aspects of children’s rights.

The theory of vulnerability is relied upon to emphasize the universal and entrenched nature of human dependency. Children are particularly vulnerable when we consider the context in which they often live and explore their world. Vulnerability theory offers an insight into how the law can help children to navigate worlds that are challenging and fraught. Fineman expands upon our understanding of this vulnerability. According to her, ‘vulnerability is—and should be understood to be—universal and constant, inherent in the human condition’.^21^ She also states that:

While all human beings stand in a position of constant vulnerability, we are individually positioned differently. We have different forms of embodiment and also are differently situated within webs of economic and institutional relationships. As a result, our vulnerabilities range in magnitude and potential at the individual level. Vulnerability, therefore, is both universal and particular; it is experienced uniquely by each of us. Important in regard to this particularity point is the fact that our individual experience of vulnerability varies according to the quality and quantity of resources we possess or can command. While society cannot eradicate our vulnerability, it can and does mediate, compensate, and lessen our vulnerability through programs, institutions, and structures.^22^

Vulnerability theory is not regarded as denying autonomy. It provides a basis where autonomy can be viewed as supported and socially situated. Therefore, the article argues for the mutual alignment of the two. By recognizing vulnerability, supportive and (if required) restrictive restrictions on liberty can be justified, while the relational version of autonomy means that these restrictions must reflect the child’s developing capacities. There is a need to assess the identification and justification of restrictions on liberty within a framework that emphasizes the child as a rights-holder, their evolving capacities, and the structural conditions of vulnerability that affect their particular circumstances.

## Setting the scene: Children and DoL

What happens in a care home for children is often careful and correct. The application of the procedures that ‘govern’ the movement of a child may sometimes be cold and clinical. In a familial home setting, a parent may say ‘lights out at 8 pm’, but this is often a moveable feast and is mediated by checks, storytelling, and chat. The ‘lights out’ regime in a care environment may often lack this fluidity of caring oversight, of loving laissez-faire. According to Series, institutional logistics continue to provide the backdrop to current care arrangements. She describes this as operating in the ‘shadows of the institution’.^23^ Despite formal deinstitutionalization, residential homes, unregulated placements, and emergency accommodation often mirror the hierarchical routines of large institutions.^24^ Their surveillance practices and loss of autonomy are broadly institutional in nature, even though attempts may be made to replicate ordinary, real domestic environments. The dynamics caused by this give rise to what Series calls an ‘institutional treadmill’.^25^ Once on this treadmill, individuals, including children, gravitate through restrictive settings where meaningful choice or prospects for change seem to exist only in the vaguest manner. Series’s analysis reveals that the boundary between ‘home’ and ‘institution’ has shattered.^26^ Institutional practices rise from the ashes of what are supposedly private settings. There is an erosion here at both legal and conceptual levels that destabilizes judicial attempts to differentiate restrictive care from ‘normal parental control’. This is because the living environments in which vulnerable children or those with disabilities can often operate as quasi-institutions, even though they may have an outward domesticity.^27^

Who are the children who are most likely to become subjects of DoL proceedings? It is clear that there is no single category of child who comes into the realm of DoL. The Nuffield Family Justice Observatory, which reviewed published judgments, indicates, however, that a pattern exists in relation to children who have complex needs.^28^ These children include, for example, those with autism and neurodivergence, children who have been traumatized, criminally or sexually exploited, children at risk of severe self-harm and those with mental health difficulties. Often, these proceedings involve children who were in care placements that broke down because their complex trauma or the risk of exploitation could not be managed by local services. Children’s Commissioner reports also refer to the fact that restrictions are often imposed when there is no access to specialized therapeutic placements.^29^ As a consequence of these experiences of trauma or due to structural shortages, there has been an effort by policymakers to intervene. However, these interventions are not fully suited to the demographic. For example, the decision to include older children in the LPS model relies on the adult capacity paradigm. This does not engage with the dependent nature of or the realities of children who have been traumatized. Also, the Children’s Wellbeing and Schools Act 2026 seeks to address issues related to placement availability, but it treats the problem as an administrative management crisis. These existing policy options lead to a reactive model of welfare paternalism. The CLPF can be used as a vehicle to fill the gap.

### The transposition gap: Mapping adult safeguards onto child dependency

It is very challenging to balance the welfare of a child with his or her autonomy in cases where the restrictive interventions may enter the realm of DoL. In the European context, *Storck v Germany* highlighted that the state must provide safeguards when restrictive care measures resulting in a DoL are in place for vulnerable people, including children.^30^ This recognizes that there can be a DoL even when interventions are intended to be in the individual’s best interests. Even if the restrictions are benign, necessary, and in the best interests of the person, there is still a DoL if they are not free to leave and are in a situation of prolonged control and supervision.^31^

The context of children is complicated by the fact that they are already, in many respects, supervised and controlled by those who hold parental responsibility. Children are typically subject to significant parental control. Many adults fall within the scope of the MCA’s DoLS (to be replaced by the LPS), particularly those with conditions such as dementia who are dependent on others for care and supervision. The mere fact of being subject to control or supervision cannot determine whether a DoL exists. Instead, the legal question must remain focused on the nature and degree of the restrictions imposed. It must be determined whether these cross the threshold identified in the relevant jurisprudence. For children, the MCA is directly relevant for those aged 16–17 years who lack capacity. However, the broader context still applies, such as the role of parental responsibility.

In *Re A-F (Children)*,^32^ it was held by Sir James Munby that, for the placement of a child to be regarded as ‘confinement’^33^ (in line with its understanding in *Storck v Germany*,^34^) the judge must compare ‘the restrictions to which the child in question is subject’ with those that would apply to a notional child of the same ‘“age”, “station”, “familial background” and “relative maturity” who is “free from disability”’.^35^ The difficulty identified by Sir James Munby stems from the failure to keep two separate questions distinct from each other. First, it must be determined whether the circumstances amount to a DoL, and secondly, whether a DoL actually exists. The latter question is important since Strasbourg jurisprudence clarified that a DoL relates specifically to objective confinement.^36^ Equally, the possibility that parents may provide valid consent is aligned with the question of legal authorization, but does not answer the question of whether the child is or is not deprived of liberty. Sir James Munby’s conclusion can be explained by the way in which parental responsibility has been understood by society to shape the baseline against which restrictions on children are assessed. From this understanding, it can be seen that restrictions that might constitute a DoL for an adult may not necessarily do so for a child. Such restrictions are understood to fall within the socially accepted scope of parental control. This does not mean that the concept of DoL can be eliminated in relation to children, but it does raise the threshold at which a finding of DoL will be made.

This threshold is further complicated by the use of a questionable line in court of first instance decisions, such as *Medway Council v A Father*^37^ and *East Riding of Yorkshire Council v The Mother*.^38^ In both cases, the controversial argument was made that, when restrictive arrangements are made for a child and parental consent is invoked (especially in relation to the use of section 20 CA and voluntary accommodation), the confinement has been deemed to come within the ‘zone of parental responsibility’ and is not ‘imputable to the state’, and, thus, puts the arrangement outside Article 5 ECHR’s scope. There is a serious flaw in the reasoning behind these decisions. By regarding parental consent as a tool to break the chain of state attribution, these cases involve conflating a parent’s domestic authority to make care decisions with the ECHR question surrounding state responsibility. The fact that the state is fundamentally involved as a result of care planning, regulatory oversight and the funding of local authorities is ignored. Under existing ECHR case law, the positive obligations of the state under Article 5 come into play when public authorities exert a central role in establishing or maintaining the conditions of confinement. The state cannot abdicate this responsibility by treating a highly institutionalized placement solely as a private, parental arrangement. A dangerous accountability vacuum is created by leaving these decisions unchallenged. This vacuum exposes the requirement for a framework that understands that state responsibility cannot be managed or avoided by opportunistically expanding domestic parental authority.

At the Supreme Court in *Re D (A Child)*, the majority held that for a child who is 16 or 17 years old in D’s position, parental consent is insufficient to avoid a DoL under Article 5 of the ECHR because ‘it is not within the scope of parental responsibility’ to consent to his type of confinement.^39^ It can be seen that existing case law points to gaps, uncertainties, imbalance and, perhaps, unfairness in the application of the DoL of children. It held that, in the context of a 16- or 17-year-old child, parents could not consent to the DoL of the child on the basis of the child’s lack of capacity.^40^ Lady Hale said that there is no guarantee that holders of parental responsibility will act in the best interests of the child, and that is why Article 5 safeguards are needed.^41^ This judgment was not made in response to the issue of capacity or incapacity. Still, it was based on the reasoning that parental responsibility does not extend to authorizing confinement after the child reaches the age of 16 or 17 years. The child’s age and the limited scope of parental authority are the key factors here. Accordingly, this case confirms that, in relation to 16- or 17-year-old children, confinement cannot be authorized on the basis of parental responsibility alone. This highlights the need for Article 5 safeguards and the limits of parental authority.

A distinct analytical framework is required by children’s DoL because childhood dependency alters the way in which confinement is operated, and state responsibility is assessed. Day-to-day instances of parental control could complicate the threshold issue under Article 5, and children’s developmental capacity can also complicate assumptions in relation to autonomy and consent. Adopting an adult-oriented model results in the risk of making excessive restrictions part of normal practice. There is also an additional risk of overstating formal autonomy without sufficient attention given to children’s lived dependency.

The distinctiveness of children’s cases does not relate solely to the presence of control, but in how parental responsibility is understood to mediate or justify that control. This is conceptually separate from the question of whether a DoL arises at all. As such, nuanced approaches are required to determine when certain restrictions go beyond what would be considered normal for a child of a similar maturity and age. It is also worth noting that, as confirmed by the Supreme Court in *Re D (A Child)*, parents’ consent cannot substitute for judicial oversight, underscoring the importance of judicial oversight in safeguarding the child’s rights.

A core difficulty with current jurisprudence is the practice of collapsing the identification of a DoL into the welfare-driven justification for it. Article 5 requires that the two issues of identification and justification remain distinct. The issue of identification concerns whether the conditions of confinement are satisfied. Justification concerns whether this confinement is necessary or proportionate. When welfare considerations enter too early into the identification stage, the procedural safeguards associated with Article 5 risk being weakened.

Under section 1 CA, paramount consideration must be given to the welfare of the child. However, academics such as Feinberg and Dworkin have argued that (hard) paternalistic interventions, even when motivated by benevolence, can undermine autonomy and regard individuals not as holders of rights but as passive subjects of care.^42^ This claim, however, presupposes that children possess autonomy that is sufficiently developed to be undermined: this assumption requires clarification. In this article, autonomy is understood in a relational and developmental sense.^43^ From this vista, children may possess emerging, partial, or context-dependent capacities for self-direction. This is different from the fully realized autonomy that is typically attributed to adults. From this viewpoint, paternalistic interventions can undermine autonomy where they unnecessarily override or fail to engage with these developing capacities (eg, where a child’s preferences or decisions are disregarded without sufficient justification). But not all paternalistic interventions have this result. Paternalistic interventions may undermine autonomy where they unnecessarily take priority over a child’s developing capacities, but some interventions may also endorse autonomy where they are participatory and proportionate.

It is important to distinguish between the identification and the justification of a DoL. A central question is whether the restrictions imposed go further than what would be regarded as normal incidents of family life for a child of that age and in those circumstances. Do the restrictions on the child amount to a DoL for the purposes of Article 5 ECHR, taking into consideration the nature, duration, effect and the way in which the restrictions are implemented, in line with the language and intent of the new Supreme Court decision of *A Reference by the Attorney General for Northern Ireland*? The identification of a DoL does not depend on whether the restrictions imposed on the child are taken by private individuals or public authorities. Just because parents have parental authority does not mean that their actions cannot result in a DoL. Substantively, a child is deprived of their liberty if these circumstances of continuous control and the child cannot leave, whether or not the best interests of the child are the motivation for the measures adopted. In order for this to be justified, it is necessary to assess whether the DoL can be defended by reference to their rights, best interests, and developing autonomy, and whether it is subject to relevant safeguards.

These challenges demonstrate how the current approach operates within an unstable, responsive framework. A tension exists between welfare paternalism and children’s rights, as they relate, in particular, to participation and protection. Accordingly, the current system is fragmented, conceptually unclear and over-reliant on welfare paternalism. The treatment of children is often characterized by a failure to properly balance autonomy and protection.

## A fractured system: The Children Act 1989, inherent jurisdiction, and Article 5 ECHR

### A patchwork of protection: Jurisdictional fragmentation

The legal approach to DoL and children in England and Wales involves consideration of the law on statutory child welfare, the inherent jurisdiction of the High Court, and Article 5 ECHR. There are different authorization routes available for children who might be deprived of their liberty.

If under 18 years, the three authorization routes are: (i) section 25 CA accommodation order (for registered secure accommodation, such as secure children’s homes, young offender centres, or secure training centres); (ii) the inherent jurisdiction of the High Court; and (iii) in relation to children who are 16 or 17 years and do not have capacity, the Court of Protection.^44^ In the context of the legal basis for the DoL, there is a requirement to identify if the authorization is based on Article 5(1)(d) (educational supervision)^45^ or (1)(e) (unsound mind) ECHR.^46^ Accordingly, the current approach consists of a patchwork of routes and a lack of a unified approach in the case law.

### Statutory limits: The Children Act 1989 and the welfare paradigm

The first of these approaches is the framework under the CA, which places an obligation on LAs to protect and promote child welfare for children in need.^47^ Under section 20, LAs have a duty to provide accommodation to children who need it, but this is voluntary and requires parental or child consent, where appropriate. When a Care Order is made under section 31, the child is placed under the care of the LA. Under section 33, the LA is conferred parental responsibility. Under section 1, the court must give paramount consideration to the child’s welfare. Section 25 allows LAs to restrict the liberty of children via secure accommodation orders where statutory criteria are met (eg, where the child might injure themselves or suffer harm if kept elsewhere). Secure accommodation placements are governed by the Children (Secure Accommodation) Regulations 1991 and regulated under the Children’s Homes (England) Regulations 2015. Placements for children under the age of 13 years require prior approval of the Secretary of State in accordance with the statutory guidance and regulatory framework. Yet, where statutory provision proves inadequate, the courts have, over time, been forced to rely on the High Court’s inherent jurisdiction.

### Judicial necessity: The High Court as a system of last resort

As addressed above, due to a lack of appropriate placements, children are sometimes not placed in a regulated environment because of resource constraints. In these circumstances, the LA can seek authorization under the High Court’s inherent jurisdiction, which goes beyond the CA. In relation to child welfare, the inherent jurisdiction operates as a means of last resort, and the courts can permit measures that would, in other circumstances, constitute a DoL. The inherent jurisdiction can only be justified when no alternative exists. The Supreme Court in *Re T* explicitly referred to the ‘shortage of provision for children and young people… whose needs are such that they require special limitations on their liberty’.^48^ A DoL should only exist in a manner that is proportionate to the risk of harm and necessary. It must comply with the procedural safeguards in line with Article 5 ECHR.^49^ In *Lancashire County Council v G (No 3)*, Macdonald J *stated* that the lack of appropriate placements has rendered the system of care structurally deficient, and that this has led courts to use powers not created for routine applications ‘by necessity’.^50^ This position puts judges in a difficult position, requiring them to give legitimacy to arrangements Parliament did not endorse. It also potentially causes exceptional approaches to be normalized and extends the bounds of judicial scrutiny.^51^

Following the rise in DoL applications, procedural reforms have been introduced.^52^ As a result of this rise in DoL applications relating to children, in July 2022, a National Deprivation of Liberty Court was created by the President of the Family Division. Its purpose was to serve as a special body for judicial oversight of DoL applications brought under the court’s inherent jurisdiction. Once this pilot scheme came to an end in 2023, the arrangements were revised. New applications became subject to the National Deprivation of Liberty List.^53^ This list is supervised by the Family Division.

To tackle crossovers with the Court of Protection, additional reforms have been instigated for children aged 16–17 years. In applications involving 16- to 17-year-old children, there must be a new proceeding and not simply a continuance of the National DoL List proceeding.^54^ This might be viewed as a minor change, but it does show a shift in thinking. This approach means that cases involving 16- or 17-year-olds who lack capacity under the MCA are handled under a jurisdiction that bases decision-making on grounds of capacity, with Article 5 ECHR rights to be represented and to have the decision reviewed.^55^

The inherent jurisdiction of the High Court has made significantly more DoL Orders over recent years. Often, these involve cases where the child has serious needs (such as behavioural or psychological needs). A notable case is *Lancashire County Council v G & N & NHS England and Lancashire and South Cumbria NHS Foundation Trust*.^56^ In this case, there were no appropriate placements available to care for a 16-year-old child whom the court recognized as vulnerable and in need of protection.^57^ This case shows that, even though the judge recognized the serious vulnerability of the child, there was no suitable placement available. It therefore illustrates the systemic dysfunction in which judges must issue Orders because there is no other alternative. The law allows for DoL, but the practical implementation that enables appropriate placements is seriously lacking. Courts have stated in numerous cases that the rise in authorizations of deprivations of liberty under the inherent jurisdiction in the context of children, often in situations where there is no other alternative, highlights a broader breakdown in the state’s capacity to comply with its duties under the ECHR and the CA.^58^

In *Nottinghamshire County Council v LH, PT and LT (No 2)*, Poole J stated that the inherent jurisdiction ought not become ‘a rubber stamp to authorize the deprivation of a child’s liberty whenever the court is told that there is no other option available’.^59^ The case law on children does not always use the language of ‘least restrictive alternative’, but the authorities make clear that restrictive placements must be used as a last resort, after other, less stringent options are explored. In *Re T*, Lady Black said that where ‘there is absolutely no alternative, and where the child (or someone else) is likely to come to grave harm if the court does not act’ it can be ‘a permissible exercise of the inherent jurisdiction to authorise a local authority to place a child in an unregistered children’s home’.^60^

### Article 5 ECHR: The procedural benchmark for liberty

The final legal mechanism is Article 5 ECHR. DoLs are permitted only if they fall within one of the exceptions and are ‘in accordance with a procedure prescribed by law’. For a DoL to be allowed, the court must, following the 2026 Supreme Court judgment, carry out a multifactorial assessment of the nature, duration, effects and manner of implementation of the DoL. It is still relevant to consider whether the restrictions result in continuous supervision where the person is not free to leave, but these no longer represent a decisive ‘acid’ test. For children, the European Court of Human Rights (ECtHR) has stated that, when the liberty of a child is at stake, there must be relevant procedural protections, such as prompt judicial review, legal representation and the ability to challenge whether the decision was legal.^61^ As affirmed in *J v Bath*, in the context of a child in care and subject to a Care Order, ‘the State can never give valid consent’ to a DoL and ‘must be afforded the benefits of checks and safeguards under Art 5(1), or… access to a process in court under Art 5(4)’.^62^ From a procedural perspective, courts must provide a review/authorization of the deprivation.^63^ Substantively, the state must have appropriate placements to give effect to the DoL orders. Therefore, it is pretty clear that the impromptu approach and/or the use of unregulated placements challenge both procedural and substantive obligations.^64^ The lack of relevant placements renders judicial oversight and authorization fictional and ineffective.

The safeguards derive from Article 5 ECHR and are designed to ensure that any DoL is lawful, necessary, and subject to appropriate procedural protections. Confinement must be authorized by a procedure prescribed by law, justified on objective grounds, and there must be access to independent and periodic review of the DoL by a court or other competent body. These safeguards also include a right to challenge the lawfulness of detention. This ensures that the individual concerned is not left solely under the control of those responsible for their care. In the context of children, these safeguards are in place to ensure that significant restrictions on liberty are not considered normal practice in familial or welfare settings without external scrutiny. This protective feature provides a critical check on the exercise of parental or state authority.

It is, therefore, apparent that an uncomfortable relationship exists among the CA, the High Court’s inherent jurisdiction, and Article 5. The CA is supposed to provide a precise statutory mechanism, and under Article 5, there is a need for vigorous procedural safeguards. However, the shortage of placements means that courts seek to bridge the gap in exercising their inherent jurisdiction. The judiciary is situated at the fault line between the CA’s intention and social reality. This shows that the law, as it stands, is limited as a system for compensating for systemic shortfalls. The result is a framework that creates an appearance that the state is complying with Article 5, while substantially failing to deliver the protections that the CA and Article 5 were designed to protect. This is an indefensible situation, and it is quite apparent that reform is needed. The law should serve as a means of protecting rights, not as a tool of crisis management.

The different legal frameworks that exist alongside one another show that cases with similar facts could be decided through different routes. This shows that a conceptual framework is needed that identifies and justifies deprivations, in order to ensure that the choice of mechanism does not obscure the nature of the restrictions imposed and the basis on which a justification exists. Therefore, a clear approach to DoL of children must operate across these different options. Identifying a DoL ought not be based on the legal route chosen, and the justification for a DoL must be based on consistent standards.

The next section examines theoretical features of the imbalance, focusing on how concepts of autonomy, welfare, and vulnerability are connected to the state’s response to the DoL of a child.

## Autonomy, capacity, and the limits of welfare paternalism

The world is hard on vulnerable children. For all sorts of reasons including, often, the need to protect them from harm, systems are put in place at institutional level or at legal level that diminish the authority these children have over their lives: in that reducing authority, a coercive force, that is often benign in intent, assumes a pervasive role in their lives. Series identifies a ‘regulatory paradox’ in which legal frameworks, whose aim is to protect vulnerable individuals, can unknowingly legitimize and entrench coercive practices.^65^ The rhetorical semantics of safeguarding can hide the power dynamics involved in care. This allows significant restrictions to be restructured as benevolent or therapeutic.^66^

The courts have, in limited circumstances, relied on the inherent jurisdiction to intervene in respect of vulnerable adults who have capacity. Adults can make decisions for themselves, even if these decisions are ostensibly unwise choices. This gives primacy to autonomy. Unlike adults, decisions made for children can often be justified on grounds of welfare and parental responsibility. However, English law does not grant parental authority absolute primacy. Under *Gillick*, if a child has ‘sufficient intelligence and understanding’, they may be deemed Gillick-competent.^67^ There is a connection between maturity and choice, but it can be challenging to reach, and its potential misapplication has been criticized in later cases. The threshold can be almost unattainable, especially if there is a serious risk of harm.^68^ In fact, the conventional wisdom is that a child’s decision to refuse treatment (even if *Gillick*-competent or lacking capacity at 16–17 years) can still be vetoed by parents or the courts.^69^

Autonomy and harm are arguably balanced by the ‘least restrictive alternative’ aspect of the MCA 2005, which says that anyone making a decision must ensure that it restricts the individual’s freedoms and rights to the least restrictive extent. The MCA applies only to people who are aged 16 years and over who lack capacity. It comes into play when an adult lacks decision-making ability. It provides a structured regime, including the authorization of a DoL when it is deemed to be necessary and proportionate to the person’s best interests. Such deprivations now fall under the mantle of the LPS (and will apply to 16- to 17-year-olds). As things stand, children are not subject to the DoLS, and 16- and 17-year-olds are in a hybrid position.^70^ The function of the CA is notably different. It is welfare-based rather than capacity-based, and best interests are paramount here. Restrictions on liberty under the CA often arise from Care Orders and the inherent jurisdiction of the High Court, even when children have decision-making ability. The MCA is ultimately linked to concepts of incapacity and to perceptions of autonomy. The CA has a broader protective jurisdiction and can authorize the same level of restriction of liberty as the MCA without the need to meet the threshold requirements of incapacity. The lack of congruence between the two regimes creates complexities, particularly for older children. The boundary between the two regimes and the proper legal pathway to be followed is not always clear-cut.

The MCA, thus, does not provide the governing legal framework for all children: its inclusion here serves solely to illustrate the principle that interferences with autonomy and liberty should be minimized and proportionate. The decision-making of younger children falls under the remit of the CA and, where applicable, the inherent jurisdiction. These two mechanisms do not contain any ‘least restrictive requirement’. At the same time, similar conditions arise through welfare analysis and the proportionality requirements under Article 5 ECHR. The MCA can, therefore, become a significant comparative measure when justifying restrictive interventions in the case of children.

## Vulnerability and parity of participation

The DoL of children is conceptually obscured by the way in which control is normalized. Practices that would clearly amount to DoL in adults are regarded as ‘normal’ for children because of parental control and the way in which welfare systems operate. Measures that protect children can impose severe restrictions on their liberty so that children can be free from harm, exploitation and self-injury. The law, as it stands, struggles to reconcile protection with respect for children as rights-holders. The legal framework currently in place inadequately distinguishes between identifying and justifying DoL. In this regard, courts frequently conflate whether a child is deprived of liberty with whether that deprivation is justified or authorized. This conflation essentially weakens legal safeguards and potentially obscures the rights of the child. The law often assumes that either children lack autonomy or that the autonomy of the child can be applied without any modification. Neither of these approaches captures in its entirety the child’s developing and relational autonomy.

### Vulnerability theory and paternalism: The twain can meet

Equally relevant is Fineman’s vulnerability theory, which considers vulnerability to be a universal human condition.^71^ There is a consequent duty on the state to instil ‘responsive’ systems that do not exploit vulnerability.^72^ According to her, there is a need for ‘responsive structures whereby state involvement actually empowers a vulnerable subject’.^73^ However, Lundy argues that the adult perspective on children as fundamentally vulnerable can actually reinforce or emphasize that vulnerability.^74^ She contends that ‘vulnerability should not eclipse agency’, because the protective assumptions of adults can result in children’s exclusion from decision-making about their own lives.^75^ She states that ‘the more vulnerable the child, the more likely it is that they will be excluded from involvement in the decisions that impact on them, with adults readily taking decisions for them, purportedly in their best interests’.^76^

There is currently too much reliance on judicial authorization instead of on a systemic rights-based mechanism. Lundy has undertaken empirical research, giving examples of the paradox. For example, she cites children with learning difficulties and explains that the transport authority painted school buses bright yellow to improve safety and visibility.^77^ What transpired is that children felt that this made them feel like subjects of ridicule and stigma.^78^ Essentially, Lundy shows through this example that the adult perspective on protection led to harm.^79^ This is also apparent in relation to DoL, because motivations that might be laudable and protective can perpetuate a cycle of imposed vulnerability and remove the perspectives of the children themselves. This example does not illustrate that paternalism is inherently unjustified. Instead, it shows that paternalism can be poorly implemented or based on flawed assumptions about welfare. The failure of a particular paternalistic intervention does not mean paternalism is, in itself, invalidated as an approach that might be adopted. In the same way, the failure of a rights-based claim does not invalidate rights discourse.

### Relational foundations: Reframing autonomy through universal dependency

In examining vulnerability theory, Grear focuses on its structural and relational elements. She criticizes individualism and argues in favour of a relational interpretation of rights.^80^ Scully claims that ‘the connections between vulnerability and loss of autonomy, using a relational approach to challenge the consensus that dependencies and their attendant vulnerabilities are incompatible with full autonomy.’^81^ She argues in favour of a version of ontological vulnerability that includes ‘the vulnerabilities that develop through all forms of dependency.’^82^ Under this interpretation, autonomy is inherently linked to support structures and relationships. This viewpoint shares parallels with how Tobin interprets the CRC.^83^ Tobin states that the CRC ought to be viewed as the realization of relational rights. From this perspective, the responsive state does not withdraw from its regulatory or protective functions. Instead, it enables children (and sometimes their families) to participate in the decision-making process by supporting the development of this decision-making capacity. It also facilitates engagement with legal and institutional frameworks. This includes enabling the expression of preferences and facilitating engagement with legal and institutional frameworks. Protection and regulation, which are still the responsibility of the state, are put into operation in a manner that tries to facilitate, instead of replacing, the voice of the child.^84^

According to Fineman, there is a positive obligation on the state to establish institutions that mitigate this vulnerability.^85^ This idea is connected to arguments made by Dworkin and Feinberg that autonomy is undermined by paternalism (which may include substituted judgment).^86^ According to Dworkin, ‘[a]utonomy is a richer notion than liberty, which is conceived either as mere absence of interference or as the presence of alternatives. It is tied up with the idea of being a subject, of being more than a passive spectator of one’s desires and feelings’.^87^ Fineman does not reject autonomy itself in *The Autonomy Myth*.^88^ Instead, she rejects the way it is conceived as a self-sufficient ideal. From the lens of vulnerability theory, autonomy is understood as something that is relational and institutionally constituted. The capacity to act autonomously is shaped by access to and availability of social, legal and material supports. Vulnerability theory gives weight to autonomy as a capacity that is enabled and sustained through responsive institutional arrangements. Vulnerability does not displace autonomy, but reframes it.

### Towards a responsive state:  Realizing participatory parity

Fraser contends, based on her principle of parity of participation, that ‘justice requires social arrangements that permit all… members of society to interact with one another as peers’.^89^

In relation to the DoL of children, Fraser’s concept of parity of participation shows that institutional constraints concerning care and decision-making can impede their capacity to participate as peers. This raises concerns about the justice and democratic legitimacy of the processes that govern their lives.

There is a link between Fraser’s parity of participation and Fineman’s responsive state. They can both be practically expressed in Article 12 of the CRC. This allows the philosophical discussion of vulnerability to be connected to procedural rights that give children a voice in decisions that affect their liberty. Fineman’s argument moves legal theory from the abstract to the recognition of universal dependency and the obligation of the state to establish responsive institutions.^90^ But Lundy is more critical of the application of the concept of vulnerability to children in a universal manner. She claims that assumptions based on paternalism can reduce the participation of children.^91^ Fineman’s perspective shows that the state has a duty to ensure that social institutions are structurally resilient.^92^ Lundy’s empirical research argues that adult-focused versions of protection can result in vulnerability.^93^ In consideration of both Fineman and Lundy’s arguments, it is apparent that a clash exists between individual agency and structural responsibility. Any reform model needs to navigate this tension. This article attempts to reconcile the responsive state, as endorsed by Fineman, with Lundy’s focus on the participatory child. The CLPF, which will be explored in detail in section ‘From theory to procedure: A case for reform and the proposed children’s liberty protection framework’, seeks to balance these variables by emphasizing participatory safeguards, such as representation, advocacy and review, within a structure that recognizes children’s dependency without equating it with a lack of capacity.

### High-risk cases and the prevention of harm

In cases involving high-risk, it is clear that the interventions might be required to prevent harm, and are justifiable, if the interventions exist within a framework that allows for serious review or scrutiny. These cases show that a framework is needed to reconcile the different variables. By adopting an approach that is not binary, the restrictive interventions can be justified while ensuring that there is a commitment to the child’s involvement in decisions that directly impact upon them.

Fineman’s vulnerability theory highlights universal dependency and provides some justification for state intervention, but it cannot be accepted without considering how it might downplay children’s agency and how overly paternalistic practices can be legitimized. Her vulnerability theory needs to be supplemented by a stricter account of rights and participation. To this end, children must be understood as rights-holders. In this regard, rights are plural and are sometimes conflicting. The rights of children in DoL contexts include liberty (Article 5 ECHR), protection, and the right of participation. These rights can conflict with one another, and the law lacks a clear framework for resolving inherent tensions. The role of the adult in the court or in the welfare system is to listen to the voice of the child and occasionally to make decisions for that child. Paternalism is not inherently illegitimate, but it must be constrained and justified. In high-risk contexts involving harm and exploitation, some paternalistic intervention is unavoidable. However, these interventions must be transparent, justified in rights-based terms, responsive to the child’s views and capacities, and subject to review and accountability. Many of the current legal safeguards are insufficiently attuned to hearing the voice of the child loudly, clearly and without judgment. The agency of the child is often a bystander on the pitch of life.

This section has highlighted an uneasy balance between harm and autonomy. For the system to be rights-based, it is necessary to reform it and rely less on judicial authorization. A framework is needed that does not simply see children as passive objects of care. They should be viewed as holders of rights whose capacities are evolving. A more rights-based mechanism is needed that gives primacy to the wishes of the child, but that also recognizes the need to consider intervening in situations involving potential harm to the child.

The argument presented here does not deny that some children need highly restrictive interventions for reasons of safety, therapeutic necessity, or protection from exploitation. The claim is made that vulnerability and welfare concerns should not dilute the procedural intensity of Article 5 protections. In fact, the greater the degree of vulnerability, the stronger the justification for robust participatory safeguards.

## From theory to procedure: A case for reform and the proposed children’s liberty protection framework

In recent years, there has been a growing understanding that an inadequate framework in relation to the DoL of children exists. For example, work by the Law Commission on DoL and social care has highlighted challenges created by the disjointed interplay of human rights frameworks, capacity and welfare.^94^ Also, the Joint Committee on Human Rights has identified issues relating to the protection of the rights of children in care settings, as well as problems regarding the wider use of confining restrictions and the adequacy of safeguards.^95^ Attempts to make improvements to oversight and the regulation of children in social care have been made following the Children’s Wellbeing and Schools Act 2026.^96^ Nevertheless, the focus of these initiatives is mainly on capacity within the system, regulation and the provision of placements. These initiatives do not create a unified statutory mechanism that can identify, authorize, and review DoL in a way that emphasizes Article 5 safeguards and child participation. It is within this context that the CLPF is proposed.

### The Children’s Liberty Protection Framework: A rights-based blueprint

There has been a significant rise in applications, suggesting that the High Court’s inherent jurisdiction is under severe pressure. There is also pressure in the social care system, which is specifically linked to the lack of specified/therapeutic placements.^97^ Sir James Munby has described this as a 'moral failure’, in which courts regularly act in a way never envisaged by Parliament.^98^ It is not merely a legal problem. The problem is multi-layered due to deficiencies in infrastructure, planning, resources, and accountability.^99^ A statutory framework that addresses these deficiencies must be created. In my view, the status quo is reactive. There is a need for the law to make vindication of rights its starting point. The CLPF proposed in this article is focused on a human rights-oriented system that puts proportionality, participation, and independent review at the core of decision-making regarding the liberty of children. The makeshift judicial system currently in place is ‘messy’, and it normalizes exceptional measures. A new statutory framework is needed. This new framework should be similar to the LPS to be adopted (which was created for adults/children over 16 years), but the new framework for children under 16 years must align with children’s developmental, social, and emotional needs.

This would allow for a procedural pathway for permitting restrictions, emphasizing the importance of proportionality and applying a ‘least restrictive alternative’ in a systematic way. The new law ought to incorporate assessments at multidisciplinary levels, periodic reviews and independent advocacy. The child’s voice should play a central role in the process. It is not only about whether there is justification for a DoL but also about ensuring that the process is reflective of the participatory vision of human rights law.

In this regard, consideration should be given to Articles 3, 12, and 37 of the CRC—best interests must be paramount, and DoL must only occur if there is no other alternative and for as short a period as possible. A new statutory regime would move away from the reactive nature of the status quo towards more proactive protection of rights. This new approach, under the suggested CLPF modelled on the LPS but child-specific, would provide greater clarity and structure to the current law. It would move towards a form of participatory parity, where the state does not emphasize control, but instead respects autonomy, human dignity, and human rights. This CLPF aims to establish a system that provides a clear statutory mechanism for granting autonomy, review, and challenge of a DoL for children under 16 years. The CLP would share similarities with the adult LPS, but its process and substance would be unique to children under 16 years.

What are the core features of the proposed CLPF? There would be a mechanism that focuses on participation and the development of the child. There would be a multidisciplinary assessment process involving psychologists, social care workers, teachers, and an independent child advocate whose job would be to ensure that the child’s views are heard and given weight. For liberty to be restricted, it would have to be necessary, proportionate, and the least restrictive alternative. An additional element would be the focus on the developmental stages of the child, his or her emotional well-being, and the willingness and ability to participate. This, therefore, differs from adult LPS (including 16- and 17-year-olds). There would be no presumption of capacity. Instead, there would be a ‘presumption of participation’. A fundamental departure from the adult LPS is marked here. The LPS relies on a linear assessment of mental capacity in order to trigger safeguards. The CLPF provides a different pathway: it prioritizes the child’s right to be heard regardless of the conceptions surrounding cognitive status. The CLPF, thus, ensures that developmental vulnerability acts as a gateway to enhanced procedural rights. It does not justify their removal.

Decision-makers would need to support the understanding and engagement of the child in the process. From a procedural point of view, there would be a periodic, independent post-authorization review conducted by the Children’s Liberty Panel, and and an appeals process before a newly devised Children’s Liberty Tribunal. This involves translating the vulnerability theory into practice. The state should be there to empower and not merely to protect citizens. This would include shifting focus from welfare paternalism to parity of participation (and, consequently, greater alignment with Articles 3, 12, and 37 CRC, as well as the Council of Europe’s recommendations in relation to children in the context of migration). In my view, the CLPF would also involve the realization of Fineman’s responsive state—procedural protections are in place that promote the voice/autonomy (and, indeed, the dignity) of the child.

The primary administrative body is the Children’s Liberty Panel. This is the administrative tier. It involves Local Authority multidisciplinary input and is chaired by an independent, legally qualified person, whose role is to ensure that the Panel does not merely ‘rubber-stamp’ social care agendas. The Children’s Liberty Tribunal has a different role. It is a superior judicial body, and its function is to provide a venue for appeals and periodic reviews. This ensures that the state’s ability to deprive a child of liberty is always based on independent judicial oversight that is aligned with the procedural rigour required by Article 5.

The main aim of the proposed CLPF is to address the statutory gap that exists for children under 16. It is also intended to eventually replace the LPS for children aged 16–17 years so that a unified, child-centred approach is created. As it stands, older children occupy a hybrid position where they are governed by an adult-based capacity model under the MCA. This model directly conflicts with the welfare jurisdiction of the CA. By bringing the 16–17 age group under the auspices of the CLPF, the ‘presumption of capacity’ is replaced with a ‘presumption of participation’. This ensures that developmental maturity and Article 12 CRC rights take precedence over linear (adult) assessments of capacity. This will end the hybrid legal status of older children. By replacing the MCA’s adult-centric model with the CLPF, the new framework establishes a liberty law for children that gives priority to developmental maturity over capacity assessments. This means that 16-year-old children and above are not compelled into an adult legal narrative that does not fit their lived reality of dependency. Under the CLPF, children of this age are given a specialized pathway that reflects the participatory spirit of the CRC. There is, therefore, a move from capacity-focused rationalism to a vulnerability-led responsive state. This represents a philosophical and legal shift towards a rights-based system that treats children up to the age of 18 years as a distinct and protected legal category.

The CLPF process starts with an identification stage. At this initial stage, the Panel conducts a child-focused multifactorial assessment. The Panel considers factors set out by the ECtHR and endorsed by the Supreme Court in *A Reference by the Attorney General for Northern Ireland*. Instead of applying the ‘acid’ test in *Cheshire West*, there would be a structured assessment undertaken by the Panel in relation to the nature, effects and implementation of the restrictions. The Panel would also consider, where appropriate, issues relating to consent and state attribution. This is separate from the justification stage. The idea is that identifying a DoL is not clouded by welfare concerns or by presumptions that restrictive approaches are justified because their purpose is to safeguard the child.

Only once a DoL is formally identified does the justification stage begin. The second phase of the process looks at the least restrictive alternative and the issue of proportionality. The child’s welfare needs are used to justify a breach of liberty. Under the framework, children subject to restrictive care arrangements would have access to independent advocacy, periodic review by an external decision-maker, participation mechanisms adapted by developmental capacity, and reason-based decision-making. These safeguards would apply in all settings, including clinical, domestic, residential, or foster.

The CLPF involves linking vulnerability and parity of participation. It is a practical example of participatory parity in operation. Under the CLPF, procedural mechanisms include the child advocate, multidisciplinary team assessments, and the Children’s Liberty Tribunal. This leads us to view the vulnerability of the child as a right to be engaged in decisions that will have a profound impact on their own liberty. We are not talking about symbolic participation. Under this model, participation is not a token gesture but a means for the state to ensure it fulfils its duty as a responsive institution. The participatory guarantees set out in Article 12 of the CRC are implemented through the procedural framework of the CLPF.^100^ Lundy contends that, for children to participate, they need an environment to present their opinions, a voice to communicate these views, someone to listen to them, and a measured influence on decision outcomes.^101^ The model of the inherent jurisdiction denies all of these. However, the CLPF would embed structured opportunities for representation, advocacy and review. It would also ensure that weight is given to the child’s actual voice, both substantively and procedurally.

A statutory framework must go beyond validating existing placements. It must actively enculture duties that maximize the child’s autonomy, examine the structural conditions that have brought about DoL, and put in place opportunities for meaningful change. Procedural change is needed. This could encompass amendments such as regular tribunal review, thorough exploration of less restorative options, and vindication of the right to advocacy. Decision-makers need to respond to the expressed wishes of the child and her long-term developmental needs. To this end, a statutory explanation is required. If the CLPF were to embed proactive scrutiny and child-centred decision-making, the failures of the existing system would not be replicated, providing a safeguard against the invidious drift towards a form of care that is, in essence, carceral, and prison-like.

### Theory in action: Archie’s case study

Let us think about a practical example and consider how Archie’s case would be dealt with under the CLPF. Archie is a 14-year-old who has multiple and complicated behavioural needs. He was placed in a very restricted residential unit. He is constantly supervised and not permitted to leave the unit. Under the status quo in England, the LA would look for High Court authorization under the inherent jurisdiction. As discussed, this is a reactive process that allows Archie very restricted rights of participation. There is also no statutory review. Instead, under the CLPF, the Panel would, in the first instance, have to follow a strict two-stage sequence. First, in the identification stage, the Panel would have to determine whether Archie’s constant supervision and inability to leave constitute a DoL as a matter of fact. The Panel would set aside his behavioural needs or the ‘good intentions’ of the staff. Only after a formal identification of DoL could the Panel move to the justification stage. It must then be proved that this specific confinement is the least restrictive alternative to meet Archie’s needs. Under this proposed framework, an independent child advocate would play a role in communicating Archie’s views. Other options, for example, could include foster placement. This would need to be explored before authorizing the restrictive placement. It would not be a decision made based on judicial discretion. Instead, the multidisciplinary Children’s Liberty Panel would make the decision about Archie. If the panel decided to approve the placement, there would be a periodic review automatically applied. This could take place every three or possibly six months. A review/appeal could also be taken to the Children’s Liberty Tribunal. Fineman’s responsive state theory would apply here. Vulnerability should not be about control. It should concern procedural fairness, accountability, and advocacy. Thus, the CLPF would turn Archie into a rights-holder whose rights are at the core of the process.

Archie’s rights have a dual function: they are both participatory and protective in nature. They include the right to express views and have those views given weight in line with maturity and age. At the same time, rights also include the right to protection from harm, including self-harm or harm arising from exploitation. In situations that involve acute risk to Archie, protective rights may justify interventions that limit the exercise of choice. This does not displace Archie’s rights as a rights-holder. Instead, there is a need to ensure that these interventions are justified, proportionate, and subject to appropriate procedural safeguards, including access to advocacy, review, and accountability mechanisms. In this way, procedural and protective rights have legal standing, and neither is sustained within paternalistic decision-making.

### The CLPF as a paradigm of procedural rights

It is contended that the CLPF could be classified as a paradigm of procedural rights. This is linked to arguments that children ought to be regarded as procedural mechanisms, in which their participation, agency, and dignity are ensured. Archard focuses on the philosophical weight given to choices made by children.^102^ Fortin argues that ‘children should be allowed to challenge any infringements of their substantive rights through the court process. Otherwise, the fact that they are rights-holders may be of little comfort to them’.^103^ These different viewpoints can be used to vindicate an argument for justice grounded in processes reflective of children’s changing capacities. The statutory framework presented in the CLPF embeds advocacy, review and participation. It regards children as holders of rights and not only as subjects of welfare. This approach, therefore, ensures that decisions are sensitive to the developmental nature of children, are fair from a procedural viewpoint and give considerable consideration to the autonomous voice of the child.

If an approach such as the proposed CLPF were accepted, it would align with Article 25 of the CRC, which calls for approaches that involve periodic review. The Independent Review of Children’s Social Care identified problems with the current system in 2022.^104^ The review criticized the reactive nature of the current law. It called for an approach that ‘prioritises relationships, consistency and accountability’ and put significant weight on the experiences of children, their involvement and accountability at a systemic level.^105^ It emphasized the rights of children, including participatory rights, and argued for systemic redesign. The independent review called for a ‘National Children’s Social Care Framework’ (NCSCF).^106^ Responding to this review, the government introduced the ‘Children’s Social Care National Framework’ (CSCNF) in December 2023.^107^ The CSCNF is focused on policy. It does not create a dedicated statutory mechanism to authorize, regulate, and review DoL for children under the age of 16 years. The CSCNF does not consider, or claim to engage with, procedural requirements under Article 5 ECHR in cases involving the DoL of children. Also, the procedural ambiguity of the status quo that relates to decision-making is not resolved by the CSCNF. The CLPF, in contrast, is a statutory model designed to address a procedural and protection-of-rights gap: the lack of a clear legal framework to authorize and review decisions involving the DoL under the age of 16 years. Thus, the CLPF implements the CSCNF’s values. These values are translated into enforceable legal duties through multidisciplinary assessment, periodic review, the Children’s Liability Tribunal, and independent advocacy. Essentially, the CSCNF is more about policy and strategy, while the CLPF is a framework of procedural rights. The CLPF is about the implementation of principles. This fills a gap regarding children under 16 years. The CSCNF pertains to what children’s social care ought to achieve. The CLPF is more about providing legal mechanisms that govern how decisions are made, reviewed or challenged.

### Beyond capacity: Why the CLPF diverges from adult safeguards

There is a divergence between the CLPF and the new LPS for adults, both in conception and design. The LPS is linked to adult-based understandings of capacity, and there is a presumption of capacity. In the CLPF, a presumption of participation exists, meaning that the child is a rights-holder. The CLPF emphasizes the developmental maturity of the child. From a procedural point of view, the ‘responsible body’ model within the LPS is replaced by the Children’s Liberty Panel. The independent child advocate is rooted in each stage of the process, and there is a right of review before a proposed Children’s Liberty Tribunal. The LPS uses best interests and capacity to assess necessity and proportionality. For the CLPF, integration includes the right to be heard under Article 12 of the CRC, the developmental well-being of the child, and the least restrictive alternative. This means that the CLPF does not involve substituted decision-making. It is about aligning the CRC’s ethos with the Council of Europe’s recommendations to create a child-oriented procedural framework.

### From abstraction to action: Putting the responsive state into practice

From a theoretical perspective, there is a shift from capacity-focused rationalism at the core of the LPS to a model based on vulnerability and parity of participation. An assumption is made in the LPS (and in DoLS) that restrictions are permissible when an adult lacks capacity to consent. From this vista, autonomy is binary, and the LPS operates by safeguarding those who lack that capacity. When it comes to the CLPF, this type of reasoning is rejected. Drawing on Fineman’s work, vulnerability is presented as a source of responsibility of the state. The LPS focuses on protection through capacity assessment, whereas the CLPF focuses on enabling children’s rights through participation.

The CLPF radically emphasizes ‘presumption of participation’ over ‘presumption of capacity’: in doing so, it recognizes that a child’s developmental vulnerability should never be a gateway to silence. In this framework, participation is embedded as a procedural right, thus affirming that all children, including those with complex needs, are viewed as being active agents in their own liberty.

It is contended that the CLPF would be in line with Article 5 ECHR, as its jurisprudence explicitly holds that decisions must be lawful, necessary, and supported by relevant safeguards. This is not currently the case under the current approach. There are duties under Articles 3, 12, and 37 of the CRC to ensure that a DoL is a means of last resort and lasts for the shortest possible period. Also, the Council of Europe’s recommendations call for independent representation and child-oriented safeguards (in the specific context of migration). The CLPF responds to these particular concerns by embedding a review-oriented, participatory approach that prioritizes the child’s development and procedural justice. This is, thus, a more coherent statutory framework that grants the child participatory rights and translates theoretical discussions of vulnerability and the responsive state into practice. This is a model of reform that bridges the gap between theory and practice around the notion that protection should not be viewed as clashing with autonomy and, instead, ought to be seen as complementary sides of the same coin.

## Conclusion

The current approach of the law, based on welfare paternalism and over-reliance on the High Court’s inherent jurisdiction, is unsuitable because it fails to ensure consistency and to promote the participatory rights of children. The current legal framework is insufficiently conceptually clear and lacks doctrinal clarity. The article has discussed the tendency to combine identification of and justification for a DoL. Vulnerability is used as a justification for control rather than to encourage empowerment. The law must be reformed, and Fineman’s vulnerability theory provides a framework for reconceptualizing the law. Vulnerability is not merely about promoting paternalism. It can also be used to justify human flourishing and empowerment, and the state should have a responsive institution that views vulnerability through this vista. It should be perceived as a universal condition that necessitates active support through procedural justice.

Vulnerability theory provides a significant lens through which to understand the conditions that result in restrictive interventions, but this must be reconciled with a developmental and relational version of autonomy. Thus, the article regards children as rights-holders, both in terms of protection and participation. By distinguishing between identification and justification, it is possible to acknowledge the realities of restrictive approaches in family and institutional environments without pre-empting the legitimacy of these approaches. This means that there can be a more structured assessment of whether they are justifiable, including in difficult cases involving serious risk of harm. By applying this framework to high-risk scenarios, there is a recognition that the autonomy of children does not prevent intervention that is protective in nature. It necessitates that these decisions are carefully considered, proportionate, and that weight is given to the child’s voice. Thus, autonomy, vulnerability and paternalism are not mutually exclusive.

The article offers a novel analysis of vulnerability as a basis for conceptualizing children’s DoL in a rights-based, less paternalistic manner. The article presents the CLPF, based on the LPS but linked to the specific, sometimes unvoiced needs and wishes of children. This article fills a practical gap by connecting theoretical discussions of vulnerability and the autonomy/protection paradox with the LPS to present a statutory mechanism for children. Thus, the article presents a model of reform that connects autonomy, protection, and vulnerability via a statutory vehicle that is coherent and promotes participatory parity.

The framework would require further development. For example, certain LAs or regional tribunals may use it as a pilot scheme. This would allow its procedural abilities and resource implications to be tested. There could also be future empirical research to help with the national implementation of the CLPF. This would also allow the implementation of the framework to be in line with key Articles of the CRC, such as Articles 3, 12, and 37 in the context of proportionality, participation, and decision-making. The focus would have to be on the state’s obligations when undertaking periodic reporting on CRC compliance. This would serve as a yardstick for policy reform and accountability.

The CLPF establishes a unified legal category for all children that protects minors from being prematurely forced into adult capacity models that do not align with their developmental reality. By establishing this framework, the law moves from a splintered approach to one that ensures that every child is regarded as a rights-holder whose liberty is protected by a single, child-centred standard of procedural justice.

The abandonment of the ‘acid’ test by the Supreme Court in *A Reference by the Attorney General for Northern Ireland* strengthens the case for the CLPF. For a DoL to be identified, there is a need for a more context-oriented approach. By recognizing the need for a holistic evaluation to identify DoL, the highest Court in the land has clearly rejected measures of confinement that are too formulistic and reductive. This, thus, represents a turning point towards a more nuanced and contextual approach that draws parallels with the CLPF’s ethos.

The article points to the procedural fragility and incoherence of the status quo, fuelled by an over-focus on judicial decision-making and too much emphasis placed on welfare to the detriment of participation and autonomy. Vulnerability is reconceptualized here as a theory for child empowerment. The CLPF presented in this article is a realization of this theoretical shift into practice, which reflects the spirit of the CRC. There is a unification of protection, autonomy and vulnerability in the CLPF. From a practical perspective, the framework identifies children as holders of rights with a voice and not merely as passive subjects. This, therefore, is a new paradigm in children’s liberty—it is about human flourishing being facilitated through the vista of parity of participation. Vulnerability has been re-evaluated as a right to participate in a transformative manner, shifting the state’s role from protection to enablement.

## Funding

This research received no specific grant from any funding agency in the public, commercial, or not-for-profit sectors.

Conflicts of interest. None declared.

## Footnotes

1 See, for example, Rosie Harding, ‘Safeguarding Freedom? Liberty Protection Safeguards, Social Justice and the Rule of Law’ (2021) Current Legal Problems 74 329.

2 ibid.

3 Lucy Series, *Deprivation of Liberty in the Shadows of the Institution* (Bristol University Press 2022) 108. Also see Ian Hacking, ‘Between Michel Foucault and Erving Goffman: between discourse in the abstract and face-to-face interaction’ (2004) 33 Economy and Society 277, 287.

4 Series ibid 107–110.

5 For a detailed discussion of procedural justice, see Denise Meyerson, Catriona Mackenzie and Therese MacDermott, *Procedural Justice and Relational Theory: Empirical, Philosophical, and Legal Perspectives* (Routledge 2021).

6 Series (n 3) 206. According to Series, ‘[h]itching safeguards to an unstable definition of “deprivation of liberty” means we are still unclear about what these safeguards are against. The courts and government increasingly cleave towards a macro interpretation of deprivation of liberty, meaning significant constraints and interferences in people’s lives may slip through the net’.

7 Recommendation CM/Rec (2019) ‘11 of the Committee of Ministers to Member States on effective guardianship for unaccompanied and separated children in the context of migration’ (Council of Europe, 11 December 2019) https://rm.coe.int/0900001680993db7 (accessed 13 November 2025).

8 [2026] UKSC 16, [184].

9 ibid [53].

10 ibid [5].

11 The timeline of this reference highlights the immediate currency of the legal shift. The Attorney General for Northern Ireland referred a devolution issue to the Supreme Court under para 34 of sch 10 to the Northern Ireland Act 1998, following continued controversy regarding the application of the *Cheshire West* ‘acid’ test in care settings. The Supreme Court, in overruling *Cheshire West* and to re-align domestic law with the jurisprudence of the ECtHR, moved the focus away from a rigid ‘acid’ test towards multifactorial assessments of the ‘type, duration, effects and manner of implementation’ of liberty restrictions.

12 ibid [4].

13 [2019] UKSC42.

14 See Jennifer Nedelsky, *Law’s Relations: A Relational Theory of Self, Autonomy and Law* (OUP 2012).

15 See Catriona Mackenzie and Natalie Stoljar (eds), *Relational Autonomy: Feminist Perspectives on Autonomy, Agency, and the Social Self* (OUP 2000); Jonathan Herring, *Relational Autonomy and Family Law* (Springer 2014).

16 Jonathan Herring, *Caring and the Law* (Hart 2014) 43.

17 Catriona Mackenzie and Natalie Stoljar, ‘Autonomy Refigured’ in Catriona Mackenzie and Natalie Stoljar (eds), *Relational Autonomy: Feminist Perspectives on Autonomy, Agency, and the Social Self* (OUP 2000) 4.

18 It is defined by Beauchamp and Childress as ‘the intentional overriding of one person’s known preferences or actions by another person, where the person who overrides justifies the action by appeal to the goal of benefiting or of preventing harm to the person whose preferences or actions are overridden’. See TL Beauchamp and JF Childress, *Principles of Biomedical Ethics* (7th edn, OUP 2012) 215.

19 Gerald Dworkin, ‘Paternalism’ (1972) 56 The Monist 64–84, 65.

20 Joel Feinberg, *Harm to Self: The Moral Limits of the Criminal Law* (OUP 1986) xvii.

21 Martha Albertson Fineman, ‘The Vulnerable Subject: Anchoring Equality in the Human Condition’ (2008) 20 Yale Journal of Law & Feminism 1, 1.

22 Martha Albertson Fineman, ‘The Vulnerable Subject and the Responsive State’ (2010) 60 Emory Law Journal 251, 268–269.

23 Series (n 3).

24 ibid 77 and 79.

25 ibid 77.

26 ibid 124–126.

27 ibid 114. According to Twigg, ‘[t]he coming of disability… may impose a new social ordering’ in domestic life’. See J Twigg, ‘The Spatial Ordering of Care: Public and Private in Bathing Support at Home’ (1999) 21 Sociology of Health & Illness 381, 381.

28 Alice Roe, ‘Children Subject to Deprivation of Liberty Orders’ (2023) *Nuffield Family Justice Observatory* <https://www.nuffieldfjo.org.uk/wp-content/uploads/2023/09/CHILDR1.1.pdf> accessed 7 June 2026; Alice Roe and Mary Ryan, ‘Children Deprived of Their Liberty: An Analysis of the First Two Months of Applications to the National Deprivation of Liberty Court’ (2023) *Nuffield Family Justice Observatory* <https://www.nuffieldfjo.org.uk/wp-content/uploads/2023/02/nfjo_report_yp_deprivation_of_liberty-1.pdf> accessed 7 June 2026.

29 Children’s Commissioner, ‘The Children Who No-One Knows What To Do With’ (2020) <https://assets.childrenscommissioner.gov.uk/wpuploads/2020/11/cco-the-children-who-no-one-knows-what-to-do-with.pdf> accessed 7 June 2026; Children’s Commissioner, ‘Children with Complex Needs Who Are Deprived of Liberty’ (2024) <https://assets.childrenscommissioner.gov.uk/wpuploads/2024/11/cc-deprivation-of-liberty.pdf> accessed 7 June 2026.

30 (2005) 43 EHRR 96.

31 ibid [37].

32 [2018] EWHC 138 (Fam).

33 ibid, see [9] and [47]

34
*Storck v Germany* (n 30).

35
*Re A-F* (n 32), [43].

36
*Guzzardi v Italy* [1980] (Appl No 7367/76).

37 [2026] EWHC 235 (Fam).

38 [2026] EWHC 81 (Fam).

39
*Re D* (n 13), [49].

40 ibid.

41 ibid.

42 Joel Feinberg, *Harm to Others* (OUP 1986); Gerald Dworkin, *The Theory and Practice of Autonomy* (CUP 1988).

43 See, for example, Jonathan Herring, *Law and the Relational Self* (CUP 2019).

44 39 Essex Chambers, ‘Guidance Note: Deprivation of Liberty and those under 18’ (January 2024) <https://39essex.com/wp-content/uploads/2024/01/Mental-Capacity-Guidance-Note-Deprivation-of-Liberty-and-under-18s-January-2024.pdf> accessed 15 May 2026.

45 ibid 8–9; *DG v Ireland* (2002) 39474/98, [80]–[85].

46 ibid; *Case of Winterwerp v The Netherlands* (1979)6301/73.

47 s 17, CA 1989.

48
*Re T* [2021] UKSC 35.

49 ibid [81]–[83].

50 [2020] EWHC 3280 (Fam), [21] and [24].

51 Note that this was also the case in Ireland before the implementation of the Special Court Order regime. It was criticized in a similar manner for operating via a means of ‘jurisdictional necessity’ that did not have suitable safeguards in the statute or consistent procedural oversight. See <https://www.lawsociety.ie/gazette/in-depth/rebels-without-a-cause> accessed 20 November 2025. Also see <https://www.childlawproject.ie/latest-volume/high-court-oversight-of-childrens-complex-care-needs/> accessed 20 November 2025.

52 See 39 Essex Chambers (n 44); Judiciary of England and Wales, ‘Revised National Listing Protocol for Applications that Seek Deprivation of Liberty Orders Relating to Children under the Inherent Jurisdiction’ (October 2023) <https://www.judiciary.uk/guidance-and-resources/revised-national-listing-protocol-for-applications-that-seek-deprivation-of-liberty-orders-relating-to-children-under-the-inherent-jurisdiction/> accessed 20 November 2025; President of the Family Division, ‘Statement on Revised National Listing Protocol for Applications that Seek Deprivation of Liberty Orders Relating to Children under the Inherent Jurisdiction’ (October 2023) <https://www.mentalhealthlaw.co.uk/media/2023-09_PFD_National_DOL_List_listing_protocol.pdf> accessed 20 November 2025.

53 ibid (*Revised National Listing Protocol*) para 1.

54 Alex Ruck Keene, ‘The Court of Protection and DoL for Those Under 18’ (*Mental Capacity Law & Policy Blog*, 2023) <https://www.mentalcapacitylawandpolicy.org.uk/the-court-of-protection-and-deprivation-of-liberty-of-those-under-18/> accessed 20 November 2025.

55 MCA 2005, s 2(5).

56 [2020] EWHC 1828 (Fam).

57 ibid [5].

58 See *Re X (A Child) (No 3)* [2017] EWHC 2036 (Fam) at [37] where Sir James Munby said that: ‘What this case demonstrates as if further demonstration of what is a well-known scandal is the disgraceful and utterly shaming lack of proper provision in this country of the clinical, residential and other support services so desperately needed by the increasing number of children and young people afflicted with the same kind of difficulties as X is burdened with.’ He also stated at [38] that: ‘The lack of proper provision for such children is a scandal which has gone on for far too long… What this case shows, as so many other cases have shown, is the utter inadequacy of the resources currently available to deal with problems of children like X.’

59 [2021] EWHC 2593 (Fam), [11].

60
*Re T* (n 48) [145].

61 See *Blokhin v Russia* [2016] ECHR 308, [196] and [219].

62
*J v Bath and North East Somerset Council and Others* [2025] EWCA 479, at [52] and [49], respectively.

63 ibid; 39 Essex Chambers (n 44) 1.

64
*Case of Winterwerp v The Netherlands* (n 46), [39].

65 Series (n 3) 231.

66 ibid, 205–206, 231.

67
*Gillick v West Norfolk and Wisbech Area Health Authority* [1985] 3 All ER 402, 410. Also see David Archard, *Children: Rights and Childhood* (3rd edn, Routledge 2015) 118.

68 See Avraham Bart, Georgina Antonia and Lynn Gillam, ‘Gillick Competence: An Inadequate Guide to the Ethics of Involving Adolescents in Decision-making’ (2024) 50 Journal of Medical Ethics 157. See Shaun D Pattinson, *Medical Law and Ethics* (5th edn, Sweet & Maxwell 2017) 165; Clayton Ó Néill, *Religion, Medicine and the Law* (Routledge 2018).

69 See *Re R (A Minor) (Wardship: Consent to Treatment)* [1992] Fam 11, *Re W (A Minor) (Medical Treatment: Court’s Jurisdiction)* [1993] Fam 64.

70 39 Essex Chambers (n 44) 4. sch 1A, MCA 2005; Mental Capacity (Amendment) Act 2019.

71 Fineman, ‘The Vulnerable Subject: Anchoring Equality in the Human Condition’ (n 21) 1.

72 ibid 19–22.

73 ibid 19.

74 Laura Lundy, ‘Vulnerability Should Not Eclipse Agency: Children’s Perspectives on Their Own Lives’ in Trude Haugli and Mona Martnes (eds), *Perspectives on Children, Rights, and Vulnerability* (Scandinavian University Press 2025) 41.

75 ibid 41.

76 ibid.

77 ibid 36–37.

78 ibid 37.

79 ibid.

80 Anna Grear, ‘Vulnerability, Advanced Global Capitalism and Co-symptomatic Injustice: Locating the Vulnerable Subject’ in Martha A Fineman and Anna Grear (eds), *Vulnerability: Reflections on a New Ethical Foundation for Law and Politics* (Routledge 2016) 41–60.

81 Jackie L Scully, ‘Disability and Vulnerabilities: On Bodies, Dependency and Power’ in Catriona Mackenzie, Wendy Rogers and Susan Dodds (eds), *Vulnerability: New Essays in Ethics and Feminist Philosophy* (OUP 2013) 206.

82 ibid 206.

83 John W Tobin, ‘Justifying Children’s Rights’ (2013) 21 International Journal of Children’s Rights 395.

84 Laura Lundy, ‘“Voice” is not enough Conceptualising Article 12 of the United Nations Convention on the Rights of the Child’ (2007) 33 British Educational Research Journal 927.

85 Martha A Fineman, ‘Equality, Autonomy and the Vulnerable Subject in Law and Politics’ in Martha Albertson Fineman and Anna Grear (eds), *Vulnerability: Reflections on a New Ethical Foundation for Law and Politics* (Routledge 2013) 17. According to her, the responsive state ‘[r]ecognises that it and the institutions brings into being through law are the mean and mechanisms whereby individuals accumulate the resilience or resources that they need to confront social, material and practical implications of vulnerability’.

86 Gerald Dworkin, *The Theory and Practice of Autonomy* (CUP 1988) 13–20; Joel Feinberg, *The Moral Limits of Criminal Law Volume 3: Harm to Self* (OUP 1989).

87 ibid (Dworkin), 107.

88 Martha A Fineman, *The Autonomy Myth: A Theory of Dependency* (The New Press 2005).

89 Nancy Fraser, ‘Social Justice in the Age of Identity Politics: Redistribution, Recognition, Participation’ (1998) SSOAR <https://www.ssoar.info/ssoar/bitstream/handle/document/12624/ssoar-1998-fraser-social_justice_in_the_age.pdf> accessed 30 November 2025. Also see: Nancy Fraser, *Justice Interrupts: Critical Reflections on the “Postsocialist” Condition* (Routledge 1997).

90 Martha A Fineman, ‘Vulnerability and Inevitable Inequality’ (2017) 4 Oslo Law Review 133, 134, 148–149.

91 Lundy, ‘Vulnerability Should Not Eclipse Agency: Children’s Perspectives on Their Own Lives’ (n 74).

92 Fineman, ‘Equality, Autonomy and the Vulnerable Subject in Law and Politics’ (n 85) 17; Fineman ‘Vulnerability and Inevitable Inequality’ (n 150) 146–148.

93 Lundy, ‘Vulnerability Should Not Eclipse Agency: Children’s Perspectives on Their Own Lives’ (n 74).

94 Law Commission, ‘Mental Capacity and Deprivation of Liberty’ (Law Com No 372, 2017) <https://cdn.websitebuilder.service.justice.gov.uk/uploads/sites/54/2025/12/Mental-Capacity-and-Deprivation-of-Liberty-Final-report.pdf> accessed 7 June 2026.

95 Joint Committee on Human Rights, ‘The Right to Freedom and Safety: Reform of the Deprivation of Liberty Safeguards’ (Seventh Report of Sessions 2017-19 (HC 890; HL Paper 161, 2018) <https://publications.parliament.uk/pa/jt201719/jtselect/jtrights/890/890.pdf> accessed 7 June 2026.

96 See Styliana, ‘Children’s Wellbeing and Schools Act 2026: Key Reforms and Initial Reflections’ (Together Trust, 6 May 2026) <https://www.togethertrust.org.uk/news/childrens-wellbeing-and-schools-act-2026-key-reforms-and-initial-reflections> accessed 7 June 2026; Adrian O’Dowd, ‘MPs reject social media ban for under 16s’ (2026) 392 BMJ 392.

97 See Steven Walker, ‘Our Children’s Services Is Broken: The Sharp Rise in Deprivation of Liberty Orders Proves It’ *The Guardian* (London, 29 May 2024) <https://www.theguardian.com/society/article/2024/may/29/our-childrens-services-system-is-broken-this-sharp-rise-in-deprivation-of-liberty-orders-proves-it> accessed 20 November 2025.

98 Louise Tickle, ‘Vulnerable Children Locked Up and ‘Gravely Damaged by the State’, Former Top Family Judge Warns’ *The Guardian* (London, 9 June 2024) <https://www.theguardian.com/society/article/2024/jun/09/locking-vulnerable-children-up-moral-failure-says-england-top-family-judge-james-munby> accessed 20 November 2025.

99 See Nuffield Justice Family Observatory, ‘Deprivation of Liberty: A Review of Published Judgments’ (NFJO 2022) <https://www.nuffieldfjo.org.uk/resource/deprivation-of-liberty-a-review-of-published-judgments> accessed 1 December 2025; National Youth Advocacy Service, ‘“The Enduring Scandal” of Pace Shortage in Secure Children’s Homes’ <https://www.nyas.net/about-us/news/new-nyas-research-report-highlights-the-enduring-scandal-of-space-shortages-in-secure-children-s-homes-for-welfare/> accessed 1 December 2025; Children’s Commissioner, ‘Illegal Children’s Homes’ (NYAS 2022) <https://www.childrenscommissioner.gov.uk/resource/illegal-childrens-homes/?utm_source=chatgpt.com> accessed 1 December 2025.

100 See Lundy, “Voice’ Is Not Enough: Conceptualising Article 12 of the United Nations Convention on the Rights of the Child’ (n 84).

101 ibid 933.

102 Archard (n 67); David Archard, ‘Hearing the Child’s Voice: A Philosophical Account’ (2020) 8 Journal of the British Academy 7.

103 Jane Fortin, *Children’s Rights and Developing Law* (3rd edn, CUP 2018) 234.

104 Independent Review of Children’s Social Care, ‘Independent Review of Children’s Social Care: The Government’s Review of the Needs, Experiences and Outcomes of Children Supported by Social Care’ (GOV.UK, 23 May 2022) <https://www.gov.uk/government/publications/independent-review-of-childrens-social-care-final-report> accessed 1 December 2025.

105 ibid.

106 ibid.

107 Department of Education, *Children’s Social Care National Framework: Statutory Guidance on Purposes, Principles for Practice and Expected Outcomes of Children’s Social Care* (Department of Education 2023).

